# Report upon the Sanitary Condition of the West Division of Chicago

**Published:** 1862-05

**Authors:** H. Webster Jones


ARTICLE XV.

### REPORT UPON THE SANITARY CONDITION OF THE WEST DIVISION OF CHICAGO, FOR THE YEAR 1860-1.

By H. WEBSTER JONES, M.D.

Read to the Chicago Medical Society, April 2, 1862.

Mr. President and Gentlemen:—As one of your Committee of Observation upon the sanitary condition of the city. I should have taken great pleasure in extending my inquiries beyond the limits of my own practice, had I possessed any earlier knowledge of my appointment, than was afforded me on Monday of the present week.

As it is, I must beg your leave to offer such brief statements and remarks upon the subject as I have been able, hurriedly, to collate from my own professional note-book, premising that my experience must not be held as a criterion upon which to base any scientific and sanitary deductions.

In fevers, I noticed a predominance of the typhoid, during the months of September, October, and November.

Most of my cases were among female children under 14 years; the disease, from its outset to the establishment of convalescence, averaging a duration of three weeks. There was in none of them any marked tendency to intestinal complication, though the gravest signs pertaining to the nervous system, were often present. As a general thing, tonics and stimulents were borne early and well. I may add, that as a remedy for continued nervous excitement and unrest, I found none equal to the cannabis indica.

I have seen few cases of intermittent or remittent fever, and those readily yielded to ordinary treatment.

Of the exanthemata, no proper epidemic has seemed to me to prevail, at any time during the year. Of scarlet fever, however, I have heard of several cases, which might be classed as "anginose," and even "malignant;" but they have proved sporadic, and were not communicated *in kind* to those who were exposed.

Measles have been frequent, but not severe, so far as I have observed.

Small pox has been *heard of*, only, during the year.

Of erysipelas I have seen no cases.

Rheumatism seemed to observe its due proportion, comparatively. In one of my cases, where the patient had, years before, suffered from a like inflammatory attack, I found instant relief to follow the use of guaiacum and colchicum, when the "alkaline treatment" had proved unavailing.

During the latter part of the winter, and early spring, I observed the prevalence of an unusually severe and obstinate influenza, attended with great congestion of the mucous linings of the nasal passages, frontal sinuses, and eustachian tubes.

I have seen a fair average, only, of pulmonary and pleuritic inflammations. Where, in one case of pleurisy, effusion took place, I found great benefit to result from the use of a strong iodine ointment, frequently applied, and protected by oiled silk and cotton, to promote its absorption.

In one case of pneumonia, in a child of eight years, I met with a rare pathological condition of the parts, a condition which might possibly, (unless the *physical signs* appertaining to it were carefully corrected by the *rational symptoms*,) lead one to error in diagnosis and prognosis.

Briefly, it was this:—At what might be considered the "height" of the disease, the lungs were both everywhere *dull under percussion*, except in their *subclavian regions*. *There* a fine crepitus was discerned; but elsewhere only bronchial respiration was audible. Within twenty-four hours after, the fine crepitus heard in the subclavian regions began to extend itself over the whole anterior surface of the lungs, which surface began also to give clearer sounds on percussion. Another day made the same facts more evident, and even posteriorly, both lungs gave the crepitus of "resolution;" but *here* the sounds elicited by the finger were still perfectly dead; at the same time, a puffiness of the neck, on the right side, was observed, which, upon pressure, distinctly crepitated. The patient steadily sank, and died in a few hours.

The autopsy, made shortly after death, disclosed a thorough hepatization of the posterior halves of the lungs, the anterior portions being emphysematous. Air had escaped into the cellular tissues of the neck, into the mediastinism, and even lay in clustered vesicles over the surface of the pericardium. The child had been comatose for forty-eight hours preceding its decease.

The point of interest was the similarity between the ordinary signs of the resolution of pulmonary inflammation, and those given by the advancing emphysema—the sounds being heard distinctly, even behind—through the condensed and hepatized structures, though, here, percussion contradicted the supposition that air was again finding its way into its accustomed channels.

Another case of pneumonia of the left lung—occurring in a boy of seven years—and advancing to the second stage of the disease, interested me in its entire and rapid disappearance under the administration of the veratrum viride alone.

During the autumn, I saw several serious cases of "diphtheritic croup," and two cases of *pure* diphtheria, which proved fatal. My impression is that the latter is a very rare disease; that it has never been as common as generally supposed; that it has not been met with so often, during the past twelvemonth, as in the preceding year; and that it has, so far as I can learn, when *distinctly* present, terminated fatally in by far the majority of cases.

During the summer months, I observed nothing unusual in regard to the prevalence or degree of severity of bowel complaints.

Since January last, I think I have heard of more cases of puerperal fevel than has been common for many months previous.

In conclusion, I believe it conceded by all, that the past year has been one of very general health, though the demands made upon our profession have been more steady, and less interrupted by periods of "stagnation," than for several years previous.

